# Patent foramen ovale closure versus drug therapy in patients over 60 years and a follow‐up of 5 years

**DOI:** 10.1002/clc.24251

**Published:** 2024-03-06

**Authors:** Angelika Eichelmann, Ralf Kubini, Dejan Nachoski, Christoph Kosinski, Michael Becker, Ali Aljalloud

**Affiliations:** ^1^ Department of Pediatrics Bethlehem Hospital Stolberg Stolberg Germany; ^2^ Rhein‐Maas Hospital, Department of Cardiology Nephrology and Internal Intensive Care Würselen Germany; ^3^ Department of Neurology Rhein‐Maas Hospital Würselen Germany; ^4^ Department of Cardiac Surgery RWTH University Hospital Aachen Aachen Germany

**Keywords:** cryptogenic stroke, elder patients, patent foramen ovale, PFO closure, PFO and atrial fibrillation

## Abstract

**Background:**

The advantages of patent foramen ovale (PFO) closure as protection from a recurrence of stroke remains controversial compared to drug therapy, especially in patients over 60 years.

**Hypothesis:**

The aim of the study is to compare recurrence of stroke in patients over 60 years old with PFO closure versus drug therapy alone.

**Methods:**

We included 342 patients over 60 years who suffered a crytopgenic stroke, and were also accepted for a PFO closure. 199 patients refused a PFO closure and were treated with medical therapy alone, whereas 143 patients underwent a PFO closure procedure.

**Results:**

The mean follow up time was 5.5 ± 1.5 years. All patients in Group B showed persistent shunt in the follow‐up period (*n* = 199, 100%). In Group A, seven patients were diagnosed with residual shunt during echocardiography examination (5%). A new onset of atrial fibrillation occurred in seven patients in Group A (5%) and six patients in Group B (3%), *p* = .117. Recurrent stroke occurred in 3 patients in Group A (2%) and 11 patients in Group B (6%), *p* = .021. One patient died of unknown reason (1%) and two patients were lost due to neurological death (1%) in Group B, whereas no patients in Group A died during the follow‐up period.

**Conclusion:**

Our results show that strict exclusion of patients over 60 years from PFO closure should be reconsidered. As life expectancies are increasing, patients should be considered for same treatment as younger patients, since the outcomes are improved compared to patients treated with medical therapy alone.

## Graphical abstract

Results of this follow‐up‐study show that strict exclusion of patients over 60 years from patent foramen ovale closure should be reconsidered. As life expectancies are increasing, patients should be considered for same treatment as younger patients, since the outcomes are improved compared to patients treated with medical therapy alone.

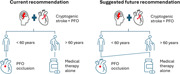

## 1 INTRODUCTION

Ischemic stroke has an estimated prevalence of 2% in western countries^1^ and remains a critical concern for public health.^2^ Cryptogenic strokes (of undetermined etiology) might cause approximately a quarter of all strokes.^3^ In the population of those afflicted by ischemic stroke, the prevalence of Patent Foramen Ovale (PFO) is significantly higher than in healthy population, especially in patients under 60 years of age (approximately 40%–45% in patients with ESUS vs. approximately 25% in healthy patients).^4^, ^5^, ^6^ A PFO is likely to cause paradoxic embolism over a right‐to‐left‐shunt.^7^ An update of nomenclature has been brought up, defining a PFO‐associated stroke.^8^, ^9^

The question of whether PFO closure in patients with cryptogenic stroke can reduce the risk of recurrent ischemic attacks, has been addressed in several studies in recent years. The first randomized studies did not show a significant positive effect of interventional PFO closure versus medical treatment alone.^10^, ^11^, ^12^

In 2017 several long‐term‐studies were published, causing a turning point in the treatment of patients <60 years of age with ESUS and PFO. Long‐term results of the RESPECT‐study, the CLOSE study and the REDUCE clinical trial all showed a relative risk reduction for ischemic events after undergoing PFO closure.^13^, ^14^, ^15^ In 2018–2019 several meta‐analysis studies followed, confirming the risk reduction for subsequent ischemic stroke after undergoing PFO closure plus medical therapy compared to medical therapy alone.^16^, ^17^, ^18^, ^19^, ^20^, ^21^, ^22^, ^23^, ^24^ International reviews and guidelines subsequently advised percutaneous PFO closure combined with medical therapy in selected patients.^10^, ^11^, ^13^, ^14^, ^15^, ^25^, ^26^

The majority of randomized controlled trials to date did not include patients >60 years of age. That is the reason why current guidelines still only recommend percutaneous closure of PFO in patients <60 years of age.^27^, ^28^

With age comes increased risk of comorbidities, which raise the incidence of venous thromboembolism, increasing the likelihood of a paradoxical embolism, making the PFO more dangerous in this patient group.^25^, ^29^ As data in this age group is still limited, further studies are needed to ensure safety and efficacy of PFO closure in older patients.^8^, ^30^

In 2020 our medium‐term follow‐up study was published, showing no significant differences in efficacy and safety after interventional closure of PFO in patients under and over 60 years of age.^31^ It was stated that patients over 60 years of age should also be considered for interventional preventive measure.

This retrospective study was performed to deliver long‐term clinical results and to show that PFO closure in patients over 60 years of age is beneficial in long‐term follow‐up.

The main emphasis lay on the comparison between patients over 60 years of age who were undergoing a preventive measure with PFO occlusion versus patients of the same age who only received medical treatment.

## 2 METHODS

### 2.1 Patients and study design

Official permission from the local ethical committee was granted. For this retrospective study, data from patients ≥60 years of age with PFO and at least one event of embolic stroke/transitory ischemic attack were collected.

PFO was diagnosed through transesophageal echocardiography examinations (TEE). All patients were examined according to thromboembolic risk factors as follows: cardiac arrythmias (electrocardiography over 72h), intrinsic small vessel disease, large vessel arteriopathy, intracardiac thrombus, masses or vegetations of the valve and hypercoagulable state (blood tests for protein C and S, antithrombin III, antiphospholipid antibodies and activated protein C resistance).

The definition of an ischemic stroke was considered as an acute focal neurological deficit, suitably caused by ischemia, that lasted for ≥24h or was connected to a relevant infarction on brain imaging (MRI, magnetic resonance imaging or CT, computed tomography as MRI‐substitute). All symptomatic patients were first seen by a neurologist, who validated the diagnosis.

Patients aged over 65 years old were divided into two groups: Group 1 included patients with cryptogenic stroke (without a known cause and without thromboembolic risk factors as listed above). These patients were divided into subgroups A and B. Group A comprised patients with cryptogenic stroke who were scheduled for PFO occlusion and Group B comprised patients who received medical treatment alone. All patients were informed of potential risks in accordance with the current guidelines that PFO can likely cause ischemic stroke and is recommended to be closed in patients under 60 years of age. Consultations were carried out by physicians not connected to this study. The assignment of patients to Groups A and B was based on patients' choice for or against PFO occlusion. Patients of Group A were scheduled for PFO closure between 2014 and 2020.

Group 2 included patients with PFO and thromboembolic stroke with thromboembolic risk factors, where the Holter ECG showed paroxysmal VHF and the carotid Doppler showed arteriosclerotic changes in the carotid artery. This Group was not examined closely for this study.

### 2.2 Diagnostics

All patients (Groups A and B) underwent transesophageal echocardiography (with Philips iE33 or GE E90) 21 ± 14 days before device implantation for baseline data as well as at 3 months and 6 months after intervention for follow‐up data. A septum primum excursion of more than 10 mm was defined as an atrial septal aneurysm (ASA).

Contrast solution was injected for shunt detection. After the opacification of the right atrium, attending physicians carried out examinations to detect microbubbles present in the left atrium during three cardiac cycles (while Vasalva maneuver or at ease). Shunts were divided by the extent to which bubbles were present: small (1–9 bubbles), moderate (≤30 bubbles) and large shunts (>30 bubbles). During follow‐up examinations, contrast echocardiography examinations were performed to ensure device position and to exclude thrombotic formations.

### 2.3 Intervention procedure in group A

At least 7 days before device implantation, all patients received 75 mg of clopidogrel and 100 mg of aspirin per day. Patients also received local anesthesia for venous access through the right femoral vein. Fluoroscopy and TEE were used to ensure precise device implantation. The device used was selected according to the interventionalist's preference (Amplatzer™ *N* = 19, Cardia Ultrasept PFO™ *N* = 109, Occlutec™ *N* = 15). The physician responsible for device implantation determined both type and size of device. The following factors influenced the decision as to which treatment was most suitable: the size of defect, whether an atrial septal aneurysm was present as well as the availability of the product and the physician's preference.

### 2.4 Post‐interventional treatment in group A

Post‐intervention, patients took 75 mg of clopidogrel per day for 3 months and 100 mg of aspirin per day for up to 9 months. Endocarditis prophylaxis was performed for 6 months. Every patient underwent TEE before being discharged from the hospital to ensure correct placement of the device. All patients were screened for post‐interventional bleeding (reduction of hemoglobin count of ≥2 mg/dl).

All study participants were clinically examined at 3 and 6 months post intervention. Afterwards, patients were contacted for annual follow‐up via telephone. The centrally recorded database was subsequently evaluated by nurses and physicians. The telephone interviews with patients or their close relatives included questions relating to appearance of cardiac death (diagnosed cardiovascular cause or death from unknown reasons, not related to cardiovascular cause) and hospitalization due to recurrent neurological symptoms or thromboembolic events. If hospitalization occured, the general practitioner responsible was consulted for further details.

96% of patients remained active in follow‐up program. 4% of patients did not respond to follow‐up consultations and were excluded from this study. Follow‐up studies took place over a mean time of 5.5 ± 1.5 years.

### 2.5 Medical treatment in group B

All patients were treated with 100 mg of aspirin per day and did not undergo interventional preventive measure with a PFO occluder.

The follow‐up program was identical to that of Group A. 94% remained active in follow‐up studies. The remaining 6% were excluded from this study.

### 2.6 Medical treatment in group 2

Patients diagnosed with atrial fibrillation were treated as a prophylaxis with direct

oral anticoagulants (DOACs), while those diagnosed with intracavitary thrombus were treated with phenprocoumon. If macroangiopathy was present, patients were treated with clopidogrel.

We excluded this group from the study.

### 2.7 Statistics

SAS‐software was selected for analyzing the results of this study. Chi‐square‐tests were performed to analyze ordinal data with a statistic significance of a *p* < .05.

Data of metric scale was stated as mean values with suitable standard deviations. The results were cross‐checked with student's *t*‐test or ANOVA.

## 3 RESULTS

### 3.1 Patient population

342 patients with PFO and cryptogenic ischemic stroke were included in this study. Group A included 143 patients who were scheduled for PFO closure (mean age 67 ± 5 years). Patients were treated with Amplatzer™ (*n* = 19), Cardia Ultrasept PFO™ (*n* = 109) Occlutec™ (*n* = 15) occluder. Afterwards patients were treated with ASS and clopidogrel for 3 months, followed by a monotherapy with ASS for 12‐24 months.

Group B included 199 patients who were not treated with PFO occlusion. Patients of this group were treated with aspirin only.

Baseline information for patients of Groups A and B are given in Table 1. Clinical and demographic features were comparable in both groups.

**Table 1:** Baseline of all included patients with mean follow‐up of 5.5 ± 1.5 years.

	Patients with PFO closure (Group A)	Patients without PFO closure (Group B)	*p*
	(*n* = 143*)	(*n* = 199)	
Age, years	67 ± 5	65 ± 6	.562
Male sex, *n* (%)	76 (53)	98 (49)	.664
Medical history, *n* (%)			
Arterial hypertension	37 (26)	48 (24)	.627
Congestive heart failure	7 (5)	6 (3)	.188
Coronary artery disease	21 (15)	48 (24)	.058
Diabetes mellitus	16 (11)	14 (7)	.411
Hypercholesterolemia	37 (26)	44 (22)	.301
Smoking	30 (21)	50 (25)	.291
Atrial septal aneurysm, *n* (%)	46 (32)	70 (35)	.281
Size of shunt by microbubbles			
Small, *n* (%)	37 (26)	44 (22)	.553
Moderate, *n* (%)	46 (32)	66 (33)	.296
Large, *n* (%)	60 (42)	89 (45)	.482

*Note*: (*Amplatzer™ *N* = 19, Cardia Ultrasept PFO™ *N* = 109, Occlutec™ *N* = 15).

### 3.2 Adverse events related to procedure

PFO occlusion was successfully performed in all 143 patients of Group A (100%). Complications occurred in 6 patients (4%) with PFO occlusion. Complications included bleeding (*n* = 3, 2%), deep vein thrombosis (*n* = 2, 1%) and infection or sepsis (*n* = 1, 1%).

### 3.3 Events during follow‐up

The mean follow‐up time was 5.5 ± 1.5 years, see Table 2. All patients in Group B showed persistent shunt in the follow‐up period (*n* = 199, 100%). In Group A, 7 patients were diagnosed with residual shunt in echocardiography (5%). A new onset of atrial fibrillation occurred in 7 patients in Group A (5%) and 6 patients in Group B (3%), *p* = .117. Recurrent stroke or TIA occurred in 3 patients in Group A (2%) and 11 patients in Group B (6%), *p* = .021. In group B, 1 patient died of unknown reason (1%) and 2 patients were lost due to neurological death (1%), whereas no patients in Group A died during the follow‐up period.

**Table 2:** Events during follow‐up (mean follow‐up of 5.5 ± 1.5 years).

	Patients with PFO closure# (*n* = 143)	Patients without PFO closure* (*n* = 199)	*p*
Cardiac death, *n* (%)	0 (0)	0 (0)	n.s.
Death of unknown reason, *n* (%)	0 (0)	1 (1)	n.s.
Device related thrombosis, *n* (%)	0 (0)	0 (0)	n.s.
**Events related to procedure, *n* (%)**	**6** (**4)**	**0** (**0)**	**.042**
Neurological death, *n* (%)	0 (0)	2 (1)	n.s.
New onset of atrial fibrillation, *n* (%)	8 (5.5)	6 (3)	.117
**Recurrent ischemic stroke/TIA, *n* (%)**	**3** (**2)**	**11** (**6)**	**.021**
**Residual/Persistent shunt, *n* (%)**	**7** (**5)**	**199** (**100)**	**.001**

## 4 DISCUSSION

Current guidelines only recommend percutaneous closure of PFO in young patients <60 years of age.^28^ Some studies indicate that patients >55 years of age benefit less from PFO closure than younger patients.^32^, ^33^ A subgroup analysis of the DEFENSE‐PFO study^25^, ^29^ showed that especially patients >70 years of age had a better outcome after PFO closure than after medical prophylaxis alone.^29^ The recurrence rate for ischemic events stays higher especially for patients >65 years of age and is likely to increase with the presence of PFO.^34^, ^35^

Medical prophylaxis alone can cause a lower quality of life in patients compared to patients undergoing PFO closure.^36^ Taking the individual life expectancy into account, a PFO closure can also be the more cost‐effective preventive measure for health systems.^37^, ^38^ Due to demographic changes, life expectancy has been increasing and should be considered before denying further treatment for all patients over 60 years of age.^39^

This long‐term retrospective study supports the results of our experiences published in 2020.^31^ Patients over 60 years of age can safely undergo PFO closure if they are carefully chosen in the first step by their attending physicians. Neurologists and cardiologist should consider previous illnesses and the current condition of each patient on a case‐by‐case basis.

Our experiences show that patients over 60 years of age benefit from PFO closure as the recurrent ischemic stroke rate was significantly lower than in patients undergoing medical preventive measure alone. In patients ≤60 years of age risk reduction for recurrent stroke is stated with approximately 55% in the biggest meta‐analysis^23^ which led to the official change in guidelines recommending PFO occlusion in younger patients. Our experiences show a relative risk reduction of 61,9% for recurrent stroke or TIA in patients ≥60 years of age. There is a minimal risk for adverse events related to the procedure, but our experiences still show an improvement of the outcome related to residual shunt and a numeric decrease of deaths of neurological cause.

As the procedure has a high level of safety so that at least one potential cause of arterial stroke can be definitively treated,^40^, ^41^ we would recommend raising the age limit and possibly also extending the indication to for example, patients with atrial fibrillation combined with a simultaneous LAA occlusion.^42^

There is a risk for adverse events related to the procedure, but our experiences still show an improvement of the outcome related to residual shunt and a numeric decrease of deaths of neurological cause.

## 5 LIMITATIONS

Our study is limited to a relatively small amount of patients and states only retrospective results and non‐randomized study, as we had to accept the patients' decision. Furthermore a recall bias is possible, as parts of follow‐up data was only collected from telephone contact with patients and physicians.

## 6 CONCLUSION

Despite the limitations, our results show that strict exclusion of patients over 60 years of age from PFO closure should be reconsidered. As life expectancy is increasing, patients should be considered for the same treatment as younger patients, since outcomes seem to improve compared to patients treated with medical therapy alone.

Further large randomized studies are needed to evidently recommend PFO closure in individuals >60 years of age and those with other reasons for arterial embolism.

## CONFLICT OF INTEREST STATEMENT

The authors declare no conflict of interest.

## Data Availability

All data are available at the Rhein‐Maas Hospital, covered by Prof. Michael Becker. The data that support the findings of this study are available on request from the corresponding author. The data are not publicly available due to privacy or ethical restrictions.
